# Recent Developments About the Pathogenesis of Dry Eye Disease: Based on Immune Inflammatory Mechanisms

**DOI:** 10.3389/fphar.2021.732887

**Published:** 2021-08-05

**Authors:** Lifei Yu, Chunjing Yu, He Dong, Yanan Mu, Rui Zhang, Qiaosi Zhang, Wei Liang, Wenjia Li, Xun Wang, Lijun Zhang

**Affiliations:** ^1^ Department of Ophthalmology, The Third People’s Hospital of Dalian, Non-Directly Affiliated Hospital of Dalian Medical University, Dalian, China; ^2^ Department of Neurosurgery, The Third People’s Hospital of Dalian, Non-Directly Affiliated Hospital of Dalian Medical University, Dalian, China

**Keywords:** Dry eye disease, immunity, inflammation, pharmaceuticals, treatment

## Abstract

Dry eye disease is a common and frequently occurring ophthalmology with complex and diverse causes, and its incidence is on the upward trend. The pathogenesis of DED is still completely clear. However, the immune response based on inflammation has been recognized as the core basis of this disease. In this review, we will systematically review the previous research on the treatment of DED in immune inflammation, analyze the latest views and research hotspots, and provide reference for the prevention and treatment of DED.

## Introduction

Dry eye disease (DED) is newly defined as eye surface disease caused by a variety of factors, tear film instability, increased osmotic pressure, ocular surface inflammation and damage, and neurosensory abnormalities play a pathogenic role, characterized by the loss of tear film balance accompanied by eye symptoms. These include dryness, foreign body sensation, burning sensation, itching sensation, photophobia, red eyes, blurred vision, fluctuating vision, and visual fatigue. In severe cases, corneal epithelial exfoliation, filamentous adhesion, and conjunctival lesions may occur (Stapleton et al., 2017). The global prevalence of dry eye disease is 5–50%, while the incidence in China is 45%, which is a high incidence area (Guo et al., 2010; Farrand et al., 2017; Stapleton et al., 2017). With the popularity of electronic products, makeup, contact lenses, environmental pollution and other influences, the number of patients with dry eye disease will continue to rise at a rate of more than 10% per year, and tend to be younger. The pathogenesis of dry eye has not yet been fully elucidated, but the eye surface immune inflammatory response as the focus of the mechanism has been increasingly concerned. In the classification of etiology, Dry Eye Workshop II regards the imbalance of tear film homeostasis as the main feature of dry eye and the core of pathophysiology, whether it is water-based tear deficiency type or over-evaporation type (Nelson et al., 2017). This process is caused by the increase of Th17 and chemokines in the ocular surface of DED patients, which breaks the normal ocular surface immune balance and leads to the immune homeostasis in the tear membrane (Kodati et al., 2014). In the clinical treatment of DED, anti-inflammatory drugs represented by cyclosporine A and immunomodulatory drugs represented by lifitegrast can play a good ameliorative effect (Wan et al., 2015; Perez et al., 2016). Therefore, it is of strategic significance to further study the immune inflammatory mechanism of DED.

## Immune Response and DED

The ocular surface immune response is a rigorous and complex regulatory process designed to protect and defend the ocular surface, but if the regulation is maladjusted, it will lead to DED (Pflugfelder and Paiva, 2017). Immune response can be divided into innate immunity and adaptive immunity. The inherent response is the innate immunity of the human body, known as the first natural defense line, which mainly includes macrophages, monocytes, dendritic cells, neutrophils and natural killer cells, etc. Adaptive immunity is acquired immunity, which generally forms a highly targeted immune process after the invasion of certain pathogenic microorganisms. The two immune modes are jointly involved in the immune regulation of dry eye disease (Schaumburg et al., 2011).

### Innate Immune Response of Ocular Surface

In the innate immunity, the human body has formed a natural physical barrier composed of sugar calyx, conjunctival epithelium, mucin, cornea, and a series of antimicrobial defense proteins in tears in the long time of survival and evolution. This barrier is a necessary part to ensure the relative safety of the eyes exposed to the external environment. After the occurrence of dry eye, the high osmotic state of patients can prevent the defense system from taking effect, and further aggravate the immune inflammatory response by directly activating the MAPK pathway to activate interleukin and tumor necrosis factor (Milner et al., 2017). At the same time, in the process of immune inflammation, Toll-like receptor signal transduction will lead to the activation of immune cells, further aggravating the inflammatory response (Lee et al., 2012).

Inflammatory immune response IIR is the most important type of innate immune response, among which macrophage and dendritic cells (DC) are common inflammatory immune cells, among which macrophages are divided into M1 and M2 cells. The former is related to cellular response, while the latter plays a regulatory role. DC are divided into myeloid cells (DC1) and lymphoid cells (DC2). In dry eye disease, DC2 cells are mainly involved in immune regulation, and the two types of cells combine with the expression of various factors, such as interleukin and tumor necrosis factor, leading to the continuous increase of inflammatory receptor levels leading to dry eye disease (Jaafar et al., 2009).

### Adaptive Immune Response of Ocular Surface

The abundant presence of CD4^+^ T cells in the adaptive immune response and cyclosporine in the treatment of DED suggest that adaptive immunity also plays an important role in DED. During the adaptive immune phase, the production of antigen-specific T cells in regional lymph nodes induces migration to the ocular surface in response to ocular stress. In this stage, the proliferation and amplification of T cells in the ocular surface cause injury, restart the acute proinflammatory innate response, and with the loss of immune regulation, trigger a vicious cycle of pathological immune response (Baudouin, 2001). DC is the most powerful known APC that can activate initial T cells, and play a dual role in the initiation and regulation of immune response, connecting innate immunity and adaptive immunity (Hackstein et al., 2016; Pan et al., 2016). In diabetes-associated dry eyes, advanced glycosylation end products can directly promote the maturation of DC and induce specific immune response of CD4^+^T cells, and the increased number and abnormal function of DC can induce adaptive immune response. (Charles et al., 2009; Surenda et al., 2011). Inflammatory factors in patients with DED can be amplified by DC, for example by upregulating the expression of TLR7 and activating the secretion of IFN-γ(Hui et al., 2010). IFN-γ induces the transformation of B cells into DC cells to produce antigen-specific antibodies, which are further activated by CD40 signaling molecules to produce large quantities of IL-6. IL-6 can promote the differentiation of Th17 cells by enhancing and inducing transcriptional activators, and secrete pro-inflammatory cytokine IL-17 to enhance immune response (Jego et al., 2003). The IL-4 secreted by mature PDC stimulates the differentiation of CD4^+^ T cells into Th2 cells, which can secrete a large number of inhibitory cytokines including IL-4, IL-10, and IL-13 upon activation. IL-4 increases B cell infiltration in lacrimal glands, thereby promoting the production of autoantibodies against acinar epithelial cells and contributing to the pathogenesis of DED (Abehsira-Amar et al., 1992).

## Immune-Based Inflammation Mechanisms in DED

Inflammation is the most common and important risk factor for DED, and studies have shown that patients with DED can detect large amounts of lymphocyte infiltration in the ocular surface and lacrimal gland tissue, at the same time, the secretion of lactoferrin decreased, the cell inflammatory factors, leading to further expansion of the scope of inflammation. Ophthalmic surface inflammation is both an initial cause and a subsequent consequence of DED (Baudouin, 2001). When the body is exposed to external stimuli or homeostasis disorders, the initiation and elimination of ocular surface inflammation are usually controlled by immunomodulatory processes. The continued inflammatory response amplifies the immune response, especially the adaptive response. This process will increase the activity of mAPC, as well as the production and recruitment of CD4^+^ Th cells in the ocular surface. When immune regulation is insufficient to eliminate inflammation or is bypassed, the activity of effector T cells will be immediately disregulated, mainly manifested by increased release of pro-inflammatory cytokines, which will lead to further inflammation and injury. The new inflammation and damage then restarts the innate immune response, creating a vicious cycle (Stern et al., 2010; Stern et al., 2013; Periman et al., 2020). At the molecular level, studies on the relationship between dry eye and inflammation mainly focus on cytokines, chemokines and signal transduction pathways.

Throughout the inflammatory response, immune cells release pro-inflammatory cytokines and chemokines, recruiting more immune cells and ultimately leading to a vicious cycle of inflammation. Current studies on inflammatory mediators associated with DED include IL-1α, IL-1β, IL-1R, IL-2, IL-4, IL-6, IL-8, IL-10, IL-12, IL-13, IL-17, IL-23, CCL-2, CCL-3, CCL-5, CCL-19, CCL-20, CCL-21, CCR2, CCR6, CCR7, CX3CL1, CXCL9, CXCL10, CXCL12, CXCR3, CXCR4, EGF, ICAM-1, IFN-γ, MMP-1, MMP-3, MMP-9, MMP-13, TNF-α, TGF-β2, VCAM-1, and NF-κB (Matsuda et al., 1998; Matsuda and Koyasu, 2000; Marsland et al., 2005; Chauhan et al., 2009; Du et al., 2009; Lam et al., 2009; Gollmer et al., 2009; Enríquez-de-Salamanca et al., 2010; Chen et al., 2011; Schaumburg, et al., 2011; Zhang et al., 2011; Zhang et al., 2012; Coursey et al., 2013; Dohlman et al., 2013; Li et al., 2013; Marko et al., 2013; Barbosa et al., 2014; Duque and Descoteaux, 2014; Ji et al., 2014; Kodati et al., 2014; Mesraoua et al., 2014; Zhang et al., 2014; Ames and Galor, 2015; Corrales et al., 2015; Contreras-Ruiz and Masli. 2015; Pierre et al., 2018; Seung et al., 2019).

IL-32 and IL-33 are new family of IL-1 cytokines involved in a variety of inflammatory diseases. A new study has found that IL-32 and IL-32-induced TSLP is a key cytokine involved in the inflammatory response through the corneal epithelial caspase-1 and NF-κB signaling pathways, providing a new molecular target for ocular surface inflammatory diseases (Jing et al., 2018). Another study found elevated IL-33 mRNA and protein levels in HCONEC cells under hypertonic conditions (Wang and Zhang, 2019). IL-33 and its receptor ST2 protein levels were higher in CIC of DE patients and correlated with the severity of the disease. In addition, the activated type 2 helper T (Th2) cells in the tears of DE patients released increased concentrations of IL-13 and IL-5, and the IL-33/ST2 pathway may play a role in the initiation of ophthalmic surface inflammation regulation. IL-33 mRNA and protein levels were increased in the corneal tissues of mice and human corneal epithelial cells (HCECs) infected with Aspergillus fumigatus. IL-33 also promoted the proliferation of HCECs cells through its receptor ST2. In addition, IL-33/ST2/p38 signaling pathway plays an important role in enhancing the inflammatory response of HCECs to Aspergillus fumigatus infection (You et al., 2019).

### Immunoregulatory Molecules and Inflammation

Corneal epithelial cells (CECs) are the main target tissues for the immunomodulatory response of DED. More and more studies have been conducted on the expression of immunomodulatory molecules in corneal epithelial cells.

Pigment epithelium-derived factor (PEDF) is a 50 kDa secreted glycoprotein with well-established anti-inflammatory functions, and then proved to be highly expressed in CECs(Ogata et al., 2002; Becerra, 2006; Zhang et al., 2006). Singh et al. found that CEPCs of mice exposed to dry stress had an amplified immunosuppressive effect on DC maturation, which was eliminated by blocking endogenous PEDF and enhanced by supplementing exogenous recombinant PEDF (Singh et al., 2020). Their subsequent experiments showed that *in vitro* culture in the presence of PEDF prevented the reduction in frequency and phenotypic inhibition of regulatory T cells induced by proinflammatory cytokines (associated with helper T cells type 17) in normal mice. Their results revealed that PEDF can promote the inhibitory ability of regulatory T cells and reduce its type 17 helper T cell-mediated dysfunction, thus playing a role in DED inhibition (Singh et al., 2021). Recently, Ma et al. found that PEDF could inhibit the expression of inflammatory cytokines IL-1β, IL-6, TNF-α, and IL-17A in DED and the percentage of Th17 cells *in vivo* and *in vitro* experiments. It was also found that PEDF inhibited the phosphorylation of MAPK p38 and JNK in hypertonic CECs(Ma et al., 2021). All the above studies have shown that PEDF plays anti-inflammatory and immunoregulatory roles in the pathogenesis of DED.

As a myxoid glycoprotein, proteoglycan 4(PRG4) is expressed in the ocular surface, which contributes to the ocular surface integrity and has a good anti-inflammatory effect (Domoto et al., 2002). Menon et al. found that HTCEPI cells synthesized and secreted PRG4, and the secretion of PRG4 was inhibited by TNFα and IL-1β *in vitro*, and exogenous rhPRG4 could significantly reduce the trend of MIP-1α and MIP-1β(Menon et al., 2021). Their experiments also found that rhPRG4 can bind to MMP-9 in human tears and inhibit the *in vitro* activity of exogenous MMP-9. *In vivo* experiments with a mouse DED model showed a significant decrease in PRG4 immunolocalization in corneal epithelium and a significant decrease in the amount of PRG4 in lacrimal gland lysate. These findings may help us to further understand the mechanism of PRG4’s immune inflammatory role in the ocular surface.

Thrombocyte reactive protein-1 (TSP-1) is a stromal cell glycoprotein first identified in activated platelets (Lawler, 1978). It can be secreted and expressed in a variety of epithelial cells (Wight et al., 1985). The secretion of TSP-1 is a protective response to inflammation, which can promote the digestion of inflammatory process and accelerate the phagocytosis of damaged cells (Doyen et al., 2003; Grimbert et al., 2006). Tan et al. found that TSP-1 mRNA expression was up-regulated in corneal epithelial cells in DED group. Compared with wild-type mice, the corneal epithelial cells of DED mice were more able to inhibit the expression of MHC-II and CD86 in DC. Moreover, topical application of recombinant TSP-1 significantly inhibited the expression of maturation of DC and proinflammatory cytokine mRNA in the mouse DED model, and improved symptoms. (Tan et al., 2018).

Programmed death ligand 1 (PD-L1) is a member of the receptor B7 family and plays a role in regulating T-cell-mediated immunity (Latchman et al., 2004). Yang et al. found that PD-L1 was highly expressed in the eye cells of DED patients, and it may control inflammation by inhibiting the production of pro-inflammatory cytokines and Th2 cytokines by activated T cells (Yang et al., 2009). EI Annan et al. found downregulation of corneal epithelial PD-L1 promotes homing of T cells to the ocular surface by increasing chemokine ligand and receptor expression, thereby amplifying DED associated keratitis and epithelial lesions. (El Annan et al., 2010).

### Autophagy and Inflammation

Autophagy is a highly conserved self-degradation process, which has been found in a variety of physiological and pathological processes in the body (Boya et al., 2016; Klionsky et al., 2021). The mechanisms involved in autophagy have been demonstrated in a variety of inflammatory diseases (Lahm and Petrache, 2012; Leung et al., 2017; Lippai and Szatmári, 2017; Rubin et al., 2017; Vij et al., 2018). Autophagy regulates inflammation by affecting the survival, development and homeostasis of inflammatory cells (Saitoh et al., 2008; Zhong et al., 2016; Qian et al., 2017), and affects the transcription, processing and secretion of inflammatory mediators (Crişan et al., 2011). Autophagy is also regulated by inflammatory factors, including IFN-γ, TNF-α, IL-1, IL-2, IL-6, and TGF-β2, which can induce autophagy, while IL-4, IL-10, and IL-13 can inhibit autophagy. The role of autophagy in DED is a research hotspot.

Recent studies of DED have shown that autophagy activation can protect the ocular surface from inflammation. Liu Zhao et al. founded that autophagy activation is a late response of HcECs to hyperosmotic stress after inflammation is triggered, which protects HcECs and promotes survival by reducing inflammatory mediators in an vitro model of dry eye. These protective effects were further enhanced when rapamycin enhanced autophagy activation in hypertonic HCECs(Liu et al., 2020a). In addition, they suggest that trehalose, as an autophagy enhancer, induces autophagy anti-inflammation by inhibiting Akt activation of transcription factor EB in primary HCECs exposed to high osmotic stress (Liu et al., 2020b). The mechanism of trehalose inhibition of inflammation is independent of NFκB pathway, and it may reduce stress-induced inflammation by inhibiting p38MAPK and activating autophagy (Panigrahi et al., 2019). Therefore, the activation of autophagy is expected to be a new strategy for the treatment of DED.

### Pyroptosis and Inflammation

Pyroptosis is a mechanism of cell death associated with the inflammatory response. Different from cell necrosis, apoptosis and autophagy, pyrodeath can be divided into classical and non-classical pyrodeath pathways. The classical pathway is induced by caspase-1, while the non-classical pathway relies on caspase-4 or caspase-5. Pyrotic cells release many inflammatory cytokines, such as IL-1β and IL-18, which trigger the aggregation of immune cells. Pyrolysis is characterized by intact nuclei, DNA strand destruction, and positive TUNEL staining (Guo et al., 2019; Liu and Sun, 2019; Yu et al., 2020) (18–21).

As a member of the immunoglobulin family, triggering receptor expressed on myeloid cells 2 (TREM2) is an immune receptor expressed on the surface of myeloid cells such as microglia, macrophages, osteoclasts and dendritic cells (Colonna, 2003; Daws et al., 2015). TREM2 may exert anti-inflammatory effects by enhancing the phagocytosis of myeloid cells (N'Diaye et al., 2009). Qu W. et al. found that compared with wild-type mice, TREM2-deficient mice were more likely to develop keratitis. This is due to the absence of TREM2 leading to increased caspase-1 and subsequent activation of cell pyroptosis and IL-1β release. In addition, caspase-1 inhibitors were found to reverse keratopathy in TREM2-deficient mice while inhibiting pyroptosis (Qu et al., 2018).

Pryrin-containing nod-like receptor protein 3(NLRP3) inflammasome is one of the inflammasomes that have been studied extensively. The production of a large number of reactive oxygen species (ROS) can activate NLRP3, and the activation of NLRP3 can activate Caspase-1, which will cause cell Pyroptosis (Mariathasan et al, 2006; Dinarello, 2009) Massive reactive oxygen species (ROS) release is the main characteristic of DED. ROS activate NLRP3 inflammasomes and lead to caspase-1 self-activation and maturation of pro-inflammatory cytokine IL-1β in a dry-eye mouse model (Zheng et al., 2014). Niu L et al. found that the mRNA and protein levels of NLRP3 were increased in patients with and without Sjogren’s syndrome, and also positively correlated with the severity of dry eye (Niu et al., 2015). The ROS-NLRP3-IL-1β signaling pathway may play an important role in the initiation of environmental induced DE models (Zheng et al., 2015). These findings suggest that NLRP3 inflammasomes may be involved in the development of ocular surface inflammation in DED.

### Apoptosis and Inflammation

Apoptosis is an active, programmed cell death controlled by genes in order to maintain internal environment stability without causing inflammatory response. In dry eyes, the apoptosis of lacrimal acinus, conjunctival epithelium, corneal epithelium, and corneal endothelial cells is abnormally increased, resulting in damage and destruction of eye tissues, while the apoptosis of lymphocytes in local tissues is inhibited, prolonging the survival time of lymphocytes and promoting the inflammatory activation state (Wilson et al., 2002; Yeh et al., 2003; Moore et al., 2011). Cysteine aspartic acid specific protease, p53 protein, and B-cell lymphoma-2 gene (Bcl-2) family proteins are involved in the signal transduction of DED cell apoptosis. Inflammation and apoptosis act together in the pathogenesis of dry eye. In the corneal and conjunctival epithelial cells of diabetic patients, the expression levels of pro-apoptotic factors such as Fas, Fasl and Bax were significantly higher than those of normal subjects, while the expression levels of anti-apoptotic factors such as Bcl-2 were relatively lower. The inflammatory environment of ocular surface can activate pro-apoptotic factors, activate apoptotic signals and activate apoptotic pathways. Moreover, the synergistic effect can further aggravate the apoptosis of corneal epithelial cells, conjunctival epithelial cells and glandular cells, leading to ocular surface abnormalities, and assist in accelerating the occurrence of dry eye disease (Hao et al., 2015). For example, in the occurrence of DED, caspase-8 and interferon-γ (IFN-γ) jointly induce and aggravate the apoptosis of conjunctival cells through the dual apoptotic pathway (X Zhang et al., 2011; X. Zhang, et al., 2014).

## List the Factors of DED Pathogenesis and Immune Inflammation

### Sex Hormone and Immune Inflammation

Epidemiological survey results show that the prevalence of dry eye disease increases significantly with age, especially in women (Cohen, 2004). Reduced androgen levels may be responsible for the higher prevalence in women than in men.

Androgen plays an important role in the pathogenesis of xerophthalmia and its mechanism is also involved in immune inflammation. Androgens have immunosuppressive effects and can maintain the balance of pro-inflammatory factors and anti-inflammatory factors in ocular surface tissues and glands, while the imbalance of androgens will increase the pro-inflammatory factors and cause eye discomfort (Babić et al., 2010; Knop and Knop, 2010; Li and Pflugfelder, 2005). The decrease of androgen level will lead to the atrophy of lacrimal epithelial cells, the disappearance of acinus mucus, the decrease of conjunctival goblet cells, the decrease of mucin expression, the shorten of tear film rupture time, and the decrease of tear quality and quantity (Sullivan et al., 2006). Androgen promotes cholesterol synthesis by regulating the gene expression of the meibomian gland, and the lack of androgen can cause damage to the lipid layer of tear film (Cohen, 2004). Androgen can down-regulate the mRNA expression of small proline rich protein, prevent excessive keratinization of the meibomian gland, and maintain the synthesis and secretion of lipid components in tears from eyelid gland (Sullivan et al., 2009).

Different opinions exist about the role of estrogen in DED and its mechanism. First of all, Zylberberg C et al. found that estrogen acted on lacrimal glands to increase the secretion of MMP-2 and 9. Suzuki T found that 17-β-estradiol up-regulated the expression of proinflammatory cytokines (IL-1,6,8) and metalloproteinases (MMP-2,7,9) in corneal epithelial cells (Suzuki and Sullivan, 2005). Studies have found that the activation of estrogen receptor B is associated with the down-regulation of the expression of enzymes required for the synthesis of serum lipoxins (LXA4) in corneal epithelial cells (Wang et al., 2011). LXA4 is a negative regulatory signal of some endogenous pro-inflammatory and pro-proliferation transmitters, which can strongly inhibit the inflammatory response *in vivo* and inhibit the chemotaxis and adhesion of neutrophils. It can be seen from the above studies that estrogen may promote ocular surface inflammation, and the increase of its level may aggravate ocular surface inflammation, which also explains that estrogen replacement therapy in postmenopausal women can not relieve dry eye symptoms, but may aggravate them. However, in another study, 17-β-estradiol was found to significantly inhibit IL-1, IL-6, and TNF-α in hypertonic corneal epithelial cells (S. B. Wang, et al., 2011). Ozcura et al. also found that 17-β-estradiol could inhibit the apoptosis of ocular surface epithelial cells (Ozcura et al., 2012). These also provide some evidence for the treatment of DED with estrogen. Therefore, the role of estrogen in DED needs to be further studied.

### Meibomian Gland and Immune Inflammation

The main function of the meibomian gland is to fight inflammation and infection. Dysfunction of meibomian gland is one of the main causes of DED. When the conjunctival epithelial cells are exposed to bacterial toxins, they can induce significant upregulation of defense genes, expression of cytokines and chemokines, TLR signaling pathway, inflammation and immune response. However, when the epithelial cells of the meibomian gland are exposed to bacterial toxins, they do not cause the expression of pro-inflammatory genes and TLR signal transduction. Thus it is speculated that the meibomian gland may have inherent anti-inflammatory and anti-infection factors (Liu et al., 2011). Omiya et al. found that Leucocyte-associated immunoglobulin-like receptor-1 expression is the highest in the meibomian gland, as a kind of inhibitory receptor, could inhibit the activation of immune cells and reduce the production of pro-inflammatory cytokines (Omiya et al., 2009). Furthermore, Leucocyte-associated immunoglobulin-like receptor-1 for differentiation of human meibomian gland epithelial cells was significantly up-regulated (Sullivan et al., 2014). Therefore, we speculate that the meibomian gland may have inherent immune anti-inflammatory and anti-infection mechanisms.

Recent studies have found that a large number of immune inflammatory cells, such as dendritic cells, were detected in corneal epithelial cells and palpebral conjunctival epithelial cells in MGD patients compared with healthy patients (Qazi et al., 2018; Zhou and Robertson, 2018). In addition, there are sex hormone receptors in the palpebral gland and palpebral cells contain enzymes necessary for endocrine synthesis and metabolism of sex hormones and steroids (Knop et al., 2011). Hampel U found that androgens can stimulate eyelid lipid secretion and inhibit inflammation, while estrogen can cause inflammation (Hampel and Garreis, 2017). As a chronic inflammatory disease, MGD induces the infiltration of a large number of immune inflammatory cells, which are important mechanisms leading to the occurrence of dry eye disease.

## Conclusion

**FIGURE 1:**
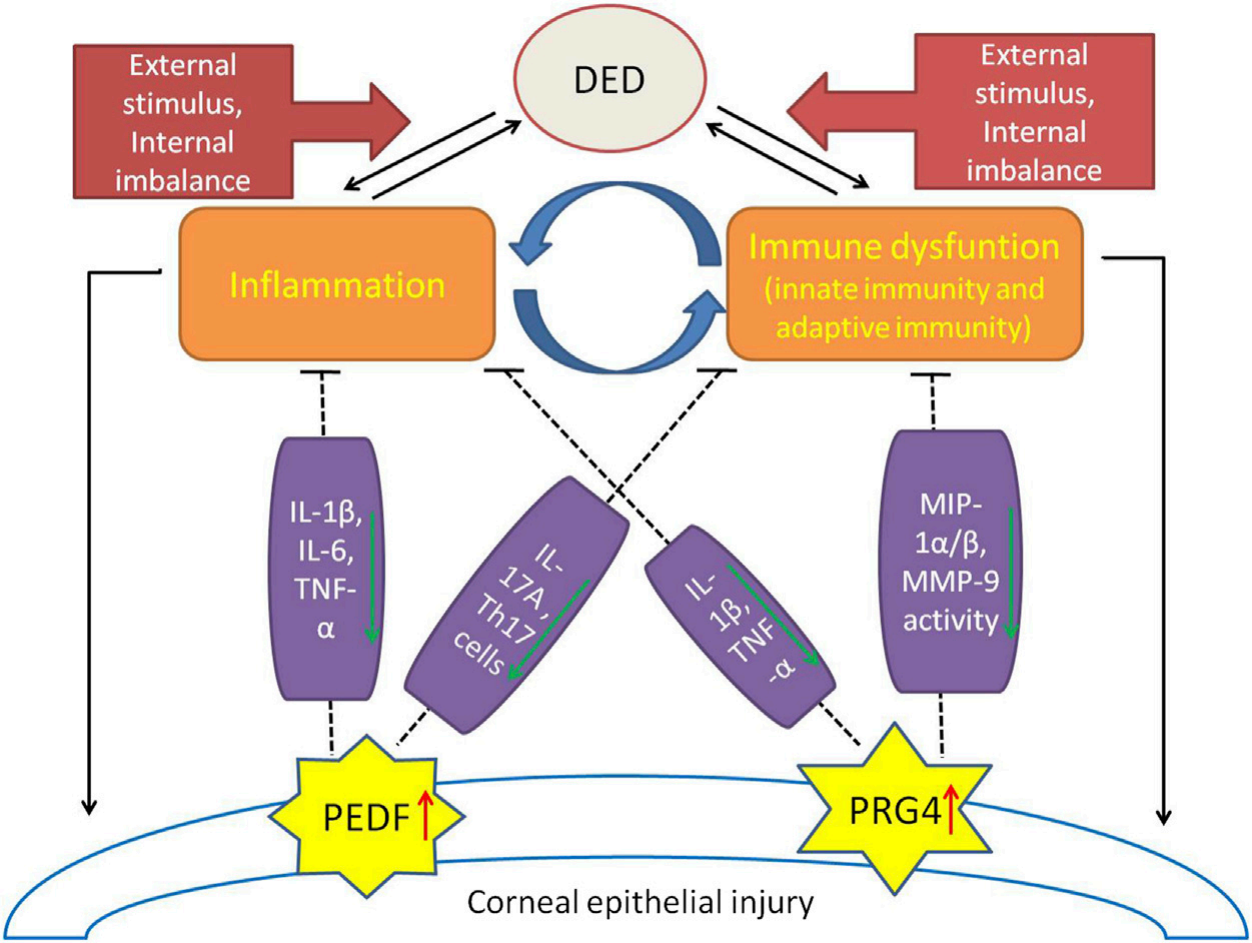
A brief view of the immune-inflammatory mechanisms in DED pathogenesis and the protective effects of immunomodulatory molecules (PEDF, PRG4). External stimulation and internal imbalance lead to the inflammatory initiation of dry eye disease and a vicious cycle of immune regulation dysfunction. Expression of PEDF and PRG4 were up-regulated in damaged corneal endothelial cells. PEDF plays a protective role in DED by inhibiting IL-1β, IL-6, TNF-α, IL-17A and the percentage of Th17 cells. PRG4 plays an immunomodulatory role in DED by down-regulating IL-1β, TNF-α, MIP-1α/β, and inhibiting the activity of MMP-9.

As the core focus of the pathogenesis of dry eye disease, immune inflammatory response has always been the focus of scholars and the research direction is relatively extensive. The most recent studies focus on the immunoregulatory molecules expressed by ocular surface cells, especially on PDEF and PRG4 (Figure 1), which have achieved exciting results. The results of these studies can not only provide new targets for the prevention and treatment of DED, but also point out a new direction for our research.
